# Minimally Invasive Surgery Combined with Regenerative Biomaterials in Treating Intra-Bony Defects: A Meta-Analysis

**DOI:** 10.1371/journal.pone.0147001

**Published:** 2016-01-19

**Authors:** Shan Liu, Bo Hu, Yuanyuan Zhang, Wenyang Li, Jinlin Song

**Affiliations:** 1 College of Stomatology, Chongqing Medical University, Chongqing, China; 2 Chongqing Key Laboratory for Oral Diseases and Biomedical Sciences, Chongqing, China; 3 Chongqing Municipal Key Laboratory of Oral Biomedical Engineering of Higher Education, Chongqing, China; 4 Wake Forest Institute for Regenerative Medicine, Wake Forest University School of Medicine, Medical Center Blvd., Winston-Salem, NC, 27157, United States of America; Bascom Palmer Eye Institute, University of Miami School of Medicine;, UNITED STATES

## Abstract

**Background:**

With the popularity of minimally invasive surgery (MIS) in periodontics, numerous publications have evaluated the benefits of MIS with or without various regenerative biomaterials in the treatment of periodontal intra-bony defects. However, it is unclear if it is necessary to use biomaterials in MIS. Thus, we conducted a meta-analysis of randomized clinical trials in patients with intra-bony defects to compare the clinical outcomes of MIS with regenerative biomaterials for MIS alone.

**Methods:**

The authors retrieved English publications on relevant studies from Cochrane CENTRAL, PubMed, Medline, Embase, Clinical Evidence, and ClinicalTrails.gov (up to June 30, 2015). The main clinical outcomes were the reduction of probing pocket depths (PPDs), gain of clinical attachment level (CAL), recession of gingival margin (REC) and radiographic bone fill. Review Manager 5.2 (Cochrane Collaboration, Oxford, England) was used to calculate the heterogeneity and mean differences of the main clinical outcomes.

**Results:**

In total, 464 studies in the literature were identified but only four were ultimately feasible. The results showed no significant difference regarding CAL gain (P = 0.32) and PPD reduction (P = 0.40) as well as REC increase (P = 0.81) and radiographic bone fill (P = 0.64) between the MIS plus biomaterials group and the MIS alone group.

**Conclusions:**

The meta-analysis suggested no significant difference in treatment of intra-bony defects between the MIS plus biomaterials group and the MIS alone group, indicating that it is important to take costs and benefits into consideration when a decision is made about a therapeutic approach. There needs to be an in-depth exploration of the induction of intrinsic tissue healing of MIS without biomaterials to achieve optimal outcomes.

## Introduction

Periodontitis, a chronic infectious disease destroying the tooth’s supporting attachment apparatus, is the leading cause of tooth loss as well as a potential hazard for the development of systematic diseases [1].To repair the damage, traditional periodontal surgery, such as open flap debridement, has often been used over the last decades [2,3]. The purpose of periodontal surgery is to reconstruct the attachment apparatus with retention or enhancement of the pre-surgical soft tissue’s contour and height. However, the results generated by traditional surgeries seemed unsatisfactory due to limited regenerated periodontal tissue. It appears that functional periodontal tissue regeneration remains a challenge. Therefore, a better surgical technique to promote periodontal *regeneration* requires further investigation.

Minimally invasive surgery (MIS), as a modern surgical procedure in a multitude of medical fields, was first introduced into the periodontal field with the intent to treat multiple and isolated periodontal intra-bony defects in 1995 [4–8]. The procedure is performed under a microscope, with microsurgical instruments and materials. It has been improved to become a minimally invasive surgical technique (MIST) with the addition of the application of gingival papilla preservation techniques [5]. Lately, a modified minimally invasive surgical technique (M-MIST) has been used as an extension of the MIST, which only elevates a buccal triangular flap [9]. The single flap approach (SFA), a novel, simplified, minimally invasive procedure, was proposed so that the unilateral mucoperiosteal flap could be elevated to retain intact adjoining soft tissues [10]. Diverse MISs have become popular and easily been accepted by periodontal practitioners and patients because of postsurgical comfort, less chair time, quicker wound healing, and lower morbidity, compared with traditional periodontal surgeries [2,11,12].

Recently, MIS has become a hot topic in research about periodontal tissue regeneration with various biomaterials, such as graft biomaterials (for example, bone graft) and growth factors (for example, enamel matrix derivative, EMD). The previous literature reported a preference for applying different types of MISs with adjunctive regenerative biomaterials to achieve favorable results in repairing intra-bony defects [4–7,9,10]. Some investigators noted that results of MIS alone were equal to that of combination with biomaterials [13–16].

It is unclear if there is any advantage to MIS combined with adjunctive biomaterials in treatment of intra-bony defects vs that of MIS alone for improvement of clinical outcomes. Hence, we conducted a meta-analysis to integrate the data and analyze the clinical efficacy of MIS plus biomaterials vs MIS alone, expecting that the result would be helpful in selection of surgical modalities to avoid unnecessary cost. In this study, we used the concept of MIS as represented by MIST, M-MIST and SFA in the following sections.

## Materials and Methods

### Search Strategy

An extensive electronic search for randomized controlled trials was conducted via the following databases: Cochrane CENTRAL, PubMed, MEDLINE (via OVID), Embase, Clinical Evidence, and ClinicalTrails.gov. The last search was updated on June 30, 2015. MeSH combined with free words was used to identify the search terms. Terms used in the search included “minimally invasive surgery”, “periodontal disease” and “intra-bony defects.” In addition, we searched the reference lists of the retrieved articles to identify any additional studies that could have been missed. Only English publications were included for screening.

### Study Selection Criteria

Using a predetermined protocol, two investigators (Shan Liu and Bo Hu) independently reviewed the studies that met the following inclusion criteria: (1) the study design was randomized and double-blinded, with a controlled clinical trial; (2) patients in the study had periodontitis, presenting at least one isolated or multiple intra-bony defect with probing pocket depths (PPDs) and clinical attachment loss (CAL) ≥5 mm; (3) in comparison studies, MIS plus regenerative biomaterials was compared with MIS alone in treatment of intra-bony defects; (4) the time frame was a follow-up that lasted at least 6 months, and the clinical parameters were evaluated at baseline and revaluated at the end of the study; (5) a full-mouth plaque score and full-mouth bleeding score of ≤25% was required; and (6) the number of patients lost to follow-up was given and reasons for the loss were explained. Patients were excluded for any of the following reasons: (1) they had systematic diseases such as diabetes mellitus, or arthritis; (2) they were pregnant or lactating; (3) they had received antibiotics, corticosteroids, or NSAIDS in the previous 3 months; or (4) they had received medical treatment that could interfere with periodontal regeneration. Any divergence between the two investigators was resolved by discussion with a third reviewer (Jinlin Song).

### Methodological Quality and Risk of Bias

All selected literature was independently assessed for the risk of bias by referring to the Cochrane Handbook for Systematic Reviews of Interventions by two investigators (Shan Liu and Bo Hu) for the following six domains: sequence generation, allocation concealment, blinding of patients and personnel, incomplete outcome data, selective reporting, and other bias. The risk of bias was assessed as follows:

1. Low risk of bias: six domains were assessed as “low risk”;
2. Moderate risk of bias: one or more domains were assessed as “unclear”;
3. High risk of bias: one or more domains were assessed as “high risk”.

### Data Extraction

For each eligible trial, two investigators (Shan Liu and Bo Hu) independently extracted relevant information using a standardized abstraction form, including the name of the first author, year of publication, study design, grouping of treatments, total participants, demographic data (mean age and sex ratio), type of intra-bony defect, number of patients lost to follow-up, duration of follow-up, mean and standard deviation (SD) of the outcome change concerning MIS with regenerative biomaterials vs MIS alone.

### Outcome Measures

Primary outcome measures:

1. Clinical attachment level (CAL) gain;
2. Probing pocket depth (PPD) reduction;
3. Recession of gingival margin (REC).

Secondary outcome measure:

Radiographic bone fill.

### Statistical Analysis

After all available data were selected and statistically pooled, two investigators (Shan Liu and Wenyang Li) performed meta-analysis using Review Manager 5.2 to yield outcomes. Mean and SD were chosen for expressing the results of continuous outcomes. Heterogeneity was evaluated through Cochrane’s test (I^2^ test) on the level of α = 0.10. If the heterogeneity was considerable (I^2^>50%), the random-effects model or subgroup analysis was employed; if the heterogeneity was non-significant (I^2^≤50%), the fixed-effects model was adopted. The statistical significance for the hypothesis test was set at p<0.05 (two-tailed Z tests). If the outcome could not be synthesized, we instead chose a descriptive analysis. Publication bias was not assessed by funnel plot in this meta-analysis owing to the small number of studies from the literature.

## Results

### Search Results

**Fig 1:**
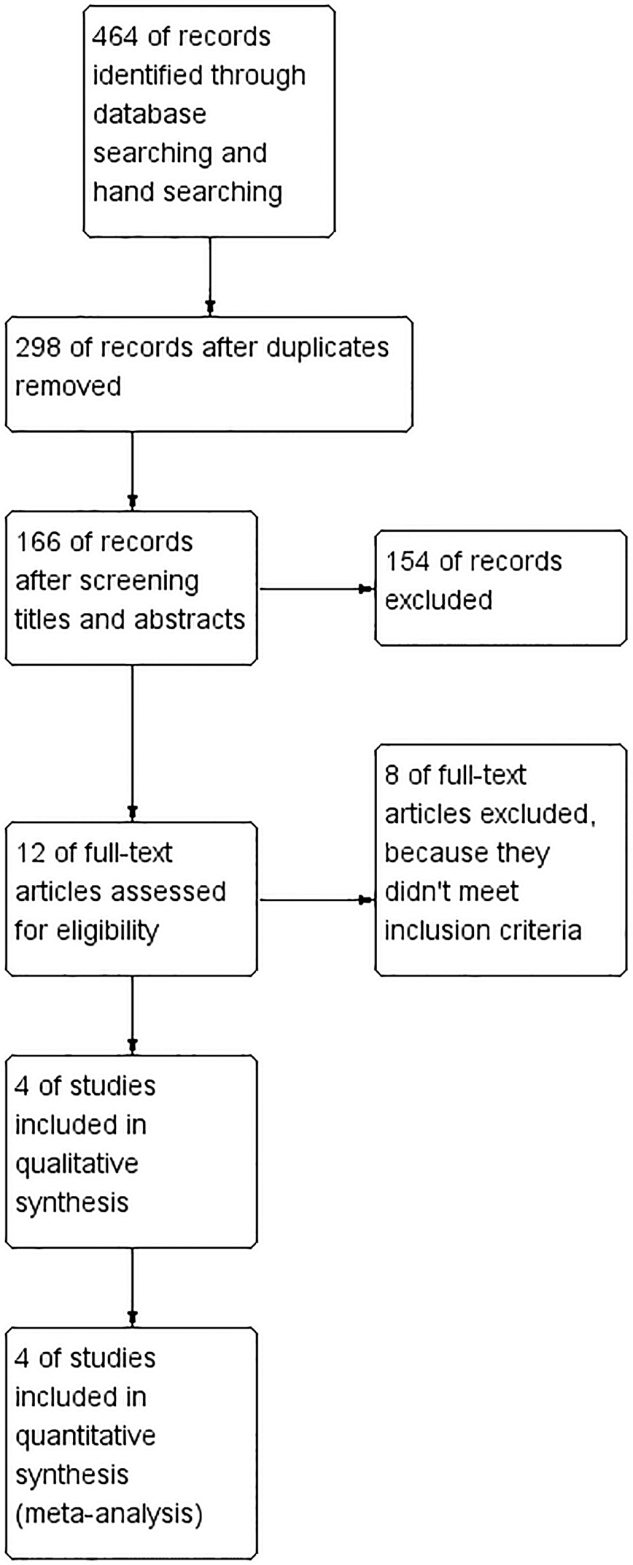
Study selection flow diagram.

Four hundred and sixty-four articles were selected through a primary database search and a hand search. After excluding 298 duplicates, 154 articles were subsequently removed by screening titles and abstracts. Eight articles were excluded after browsing the full text, and, finally, four studies from the literature that met the inclusion criteria were adopted [13–16]. The flow chart of the inclusion process is shown in Fig 1.

### Characteristics of Included Studies

Four randomized, double-blinded, controlled trials were eventually included. All included studies were of parallel design. The mean age of patients in each study was significantly different. Patients in the study of Cortellini & Tonetti [13] were followed-up for 1 year, and other three studies’ patients [14–16] were included in a 6-month follow-up. Among these studies, Cortellini & Tonetti performed a three-armed trial (Group G1 with MIST alone, group G2 with MIST combined with EMD, and group G3 with MIST combined with EMD and bone graft) [13]. Considering that different bone grafts may have an addictive influence on the results and previous studies focused on combining MIST with EMD [4–7,17,18], G1 and G3 were selected for meta-analysis. Four studies provided data approximately 108 subjects; every subject who presented at least one site for an intra-bony defect (PPD and CAL ≥5 mm) was considered as a sample for statistically analyzing the results. However, in the study by Mishra and colleagues [14], two patients were lost to follow-up because they were not able to attend the last post-surgical visit. In the study by Ribeiro and colleagues [16], they had one patient lost to follow-up owing to administration of an antibiotic medication. Hence, only 105 patient outcome variables were available to be statistically pooled. The characteristics of included studies are tabulated in Table 1 [13–16].

**Table 1:** Characteristics of included studies.

Study ID	Cortellini & Tonetti [14]	Ribeiro [15]	Mishra [16]	Trombelli [17]
	2011	2011	2013	2010
**Study Design**	RCT, parallel	RCT, parallel	RCT, parallel	RCT, parallel
**Types of Intrabony defects**	1-or 2- or 3- or combination	**-**	2- or 3-wall or combination	1- to 2- or 2- to 3- or 3-wall
**Treatment Groups**	MIST+EMD	MIST+EMD	M-MIST+rhPDGF-BB	SFA+HA/GTR
	MIST	MIST	M-MIST	SFA
**Participant Mean Age (year)**	30 participants	30 participants	24 participants	24 participants
	MIST+EMD:47.2±5 yr.	47.1±6.9 yr.	25–50 yr.	SFA:56.3±5 yr.
	MIST: 48.9±7.9 yr.			SFA+HA/GTR:46.5±8.5 yr.
**Loss Follow-up**	none	1-experiment group	2(1-experiment group,1-control group)	none
**Female (%)**	47	66	50	29
**Duration (months)**	12	3,6	6	6

**Abbreviations:** RCT: randomized clinical trial; MIST: minimally invasive surgical technique; M-MIST: modified-minimally invasive surgical technique; SFA: single flap approach.

### Methodological and Quality Assessment

**Fig 2:**
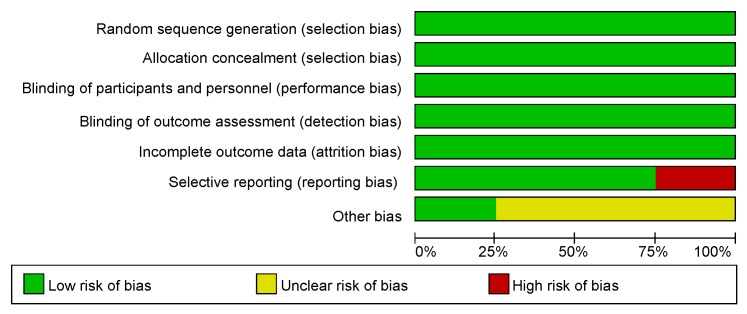
Risk of bias graph. Review authors’ judgments about each risk of bias item presented as percentages across all included studies. Red, yellow, and green refer to high risk of bias, unclear risk of bias, and high risk of bias.

**Fig 3:**
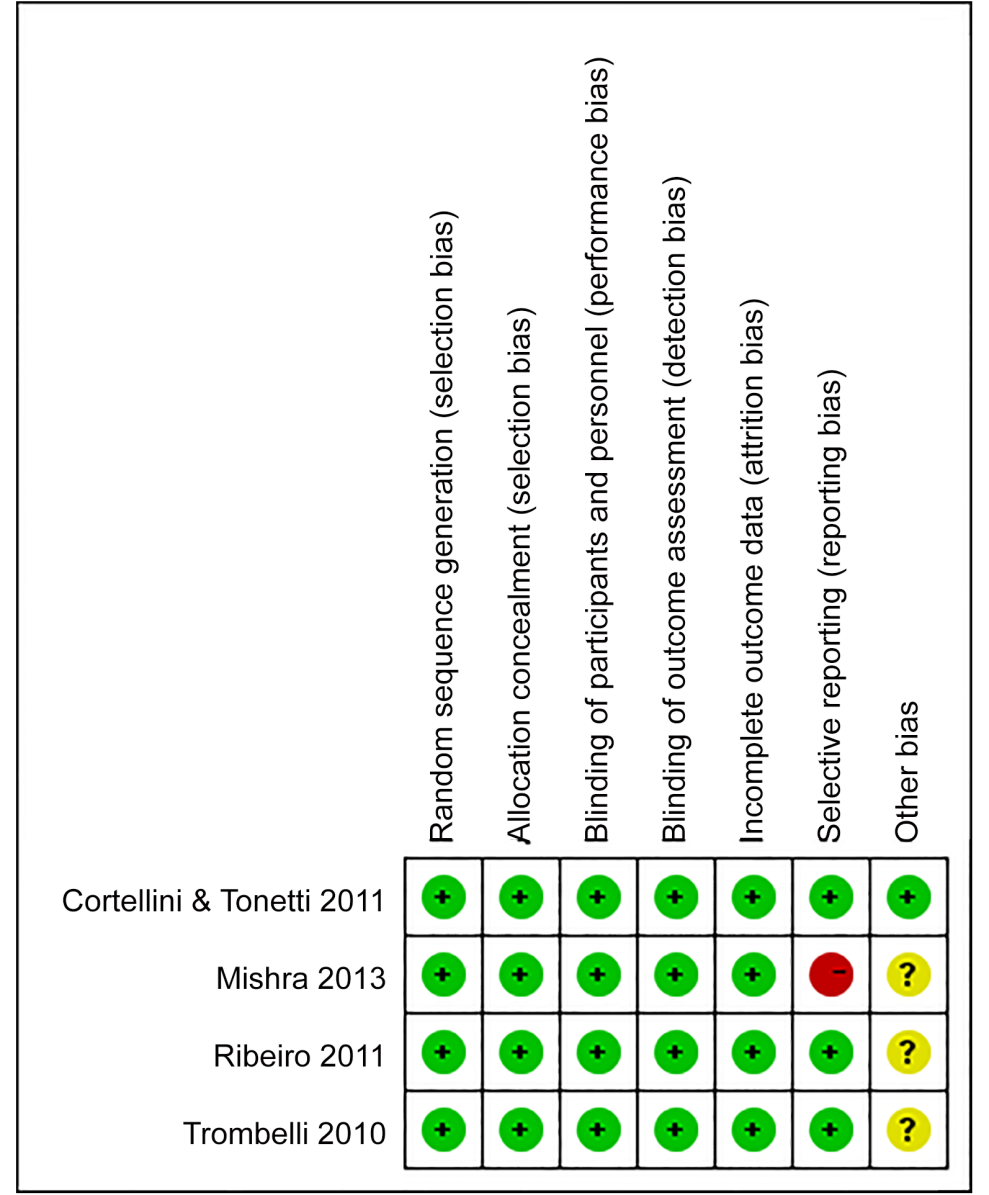
Risk of bias summary. Review authors' judgement about each risk of bias item for each included study. Red, green, and yellow refer to high risk of bias, low risk of bias, and unclear risk of bias, respectively.

Random sequence generation, allocation concealment, a double-blinded process, and a blinded outcome assessment were applied in the four studies we used. The assessment of risk of bias was related to the Cochrane Handbook for Systematic Reviews of Interventions. In the four included studies, one [13] showed a low risk of bias, two [15,16] exhibited a moderate risk of bias, and the residual study [14] presented a high risk of bias. A review of the authors’ judgment about each risk of bias item is presented in Fig 2, and the results of each study’s risk of bias are listed in Fig 3.

### Outcome Measures

We extracted feasible data, pooled them statistically, and then performed meta-analysis to determine the overall efficacy of MIS plus biomaterials and MIS alone in the treatment of intra-bony defects.

**Fig 4:**
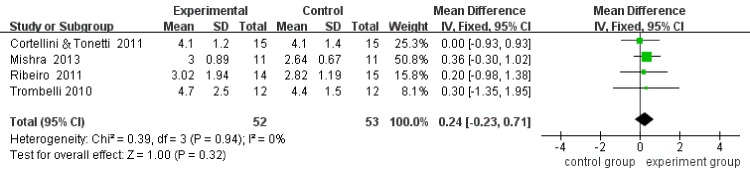
Forest plot of CAL gains in comparison of MIS with regenerative material versus MIS alone.

**Fig 5:**
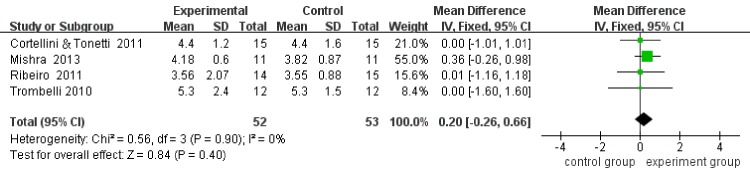
Forest plot of PD reduction in comparison of MIS with regenerative material versus MIS alone.

**Fig 6:**
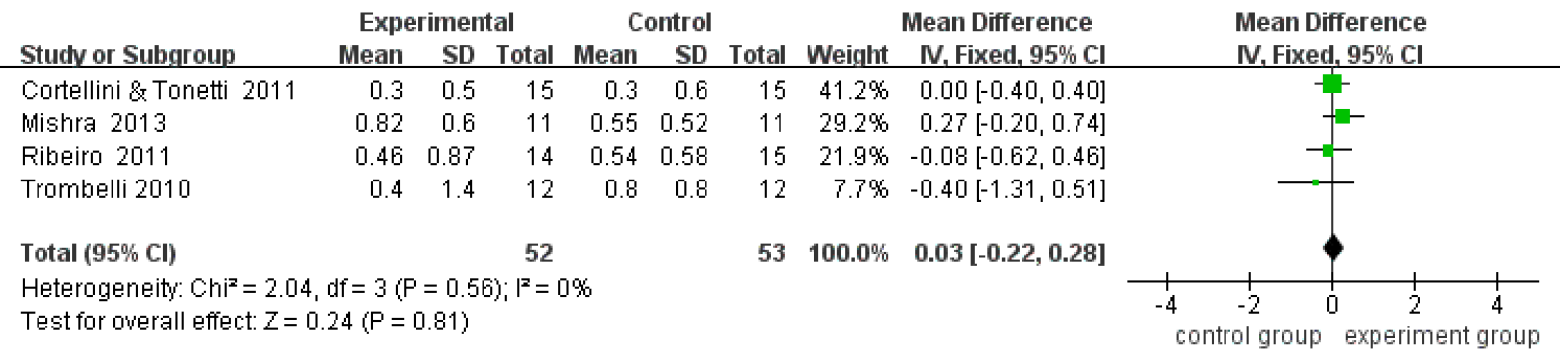
Forest plot of REC in comparison of MIS with regenerative material versus MIS alone.

**Fig 7:**
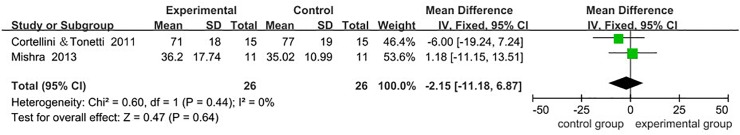
Forest plot of radiographic bone fill in comparison of MIS with regenerative material versus MIS alone.

Four included studies reported CAL, PPD, and REC at baseline and at the end of follow-up, respectively. Considering that the analysis of heterogeneity presented low (I^2^ = 0), the fix-effect model was adopted. The results revealed that the mean CAL gains in the MIS plus biomaterials group was 0.24 mm greater (95%CI:-0.32–0.71, p = 0.32) than the mean CAL gains in the MIS group. No significant differences (P = 0.32) were detected between the two groups (Fig 4). Moreover, according to the results, there was only 0.20 mm more (95%CI: -0.26–0.66, p = 0.40) PPD reduction in the MIS plus biomaterials group than in the MIS alone group, and there was no significant difference (P = 0.40) between the two groups (Fig 5). For REC, the outcome showed that the MIS plus biomaterials group contributed a mere 0.03 mm more (95%CI: -0.22–0.28, p = 0.81) REC than the MIS alone group. However, differences (P = 0.81) between the two groups were not statistically significant (Fig 6). Only two [13,14] studies detected the bone fill by radiography. After statistical pooling, the fix-effect model was chosen for the two studies because of the low heterogeneity (I^2^ = 0). The result indicated that the MIS alone group attained 2.15% (95%CI: -11.18–6.87, p = 0.64) extra bone fill than the MIS plus biomaterials group. However, no significant differences (P = 0.64) between the two groups were noted (Fig 7).

## Discussion

Minimally invasive therapeutic modalities have been successfully used for many medical procedures as the standard care. In contrast, the use of minimally invasive techniques in periodontal therapy has not progressed to the same extent [19]. As far as we know, the standard surgical procedures used to treat periodontitis have remained essentially unchanged for decades [20,21]. Due to the friendly, patient-oriented pattern and excellent clinical outcomes of the MIS procedure, MIS has been well received by both patients and periodontal practitioners as a promising surgical technique. Moreover, surgical technique of dentists can be enhanced with microsurgical instruments under a microscope. With the refinement of MIS, the average rate of primary closure of the wound in the treatment of intra-bony defects was 100% and was maintained at 95% for one week in patients with a single site defect [5,7].And the average rate of primary closure of the wound in the treatment of intra-bony defects was 100% for one week in patients with multiple sites [4,22]. In providing a robust environment to support periodontal regeneration, it was reported that MISs are superior to traditional surgeries. Furthermore, one thing deserves to be mentioned is that the final results of this study indicated that MIS should be considered as a promising surgical approach for periodontal regeneration. Meanwhile, biomaterials science is rapidly developing simultaneously with the continuous improvement of minimally invasive technique. Regenerative biomaterials (such as EMD, bone graft, etc.) have been used successfully in periodontal regeneration. Several previous case reports focused on demonstrating that the application of MIS with these biomaterials encouraged intra-bony defect reparation [4,5,7,17,18]. Nevertheless, it is more rational to center on the large success we have achieved by using MIS alone. Therefore, it is imperative to determine if the combination of MIS with biomaterials is of more use than MIS alone.

The present meta-analysis showed no statistically significant difference in all of the measured clinical outcomes between the MIS plus biomaterials group and the MIS alone group, verifying that MIS with adjunctive biomaterials did not facilitate a superior treatment of intra-bony defects. When analyzing the clinical significance of MIS with or without regenerative biomaterials, we could conclude that MIS showed an extraordinary clinical healing capacity in the treatment of intra-bony defects. This healing capacity was shown especially in terms of the result of a substantial amount of CAL gain as well as in terms of the result of a clinically significant reduction in PPDs. CAL gain and PPD reduction are frequently used as clinical parameters to demonstrate the success of periodontal treatment [23,24]. In this meta-analysis, the substantial mean increase in CAL gains ranging from 2.64 mm to 4.7 mm at the last follow-up visit largely exceeded other types of regenerative periodontal procedures [13–16,25–27]. The mean reduction of PPDs reached 3.55 mm to 5.3 mm, proving a favorable inflammation decrease in favor of periodontal regeneration with MIS [13–16].

From the view of real patient-centered outcomes, esthetics is an indispensable part that cannot be ignored, so REC was chosen as a major outcome. Generally, routine periodontal surgeries are tightly associated with recession of the gingival margin, which seems, from an esthetic point of view, an inevitably adverse sequel that impairs patients’ satisfaction [28–30]. However, the results of the mean REC presented 0.3 mm to 0.82 mm [13–16] versus 1.2 mm to 2.2 mm [28–32] for MISs and regular surgical approaches, respectively, for the treatment of intra-bony defects. This finding revealed a striking gingival margin preservation via MIS. The limited gingival recession could be a favorable consequence that meets many patients’ high esthetic demands. The distinguishing features of MIS, the retention of pre-existing papillary height and contour, might be attributed to decreased tissue manipulation, lessened overall trauma and enhanced blood supply to the surgical sites. Moreover, to reflect periodontal regeneration from a radiological perspective, we also selected radiographic bone fill as a secondary outcome measure. The radiographic images revealed a substantial amount of active bone formation in both included studies [13,14].

Meanwhile, after an in-depth analysis of the absence of significant difference, the following six aspects could explain the results of this study. (1) The resolution of intra-bony defects achieved with MIS might be a consequence of the intrinsic healing potential of the surgery. (2) The use of a microscope exerted a positive role in broadening our vision, providing ample surgical access and making manufacturing rather meticulous and precise. Therefore, management of inter-dental soft tissues facilitated blood clot stability and maturation, flap margin blood perfusion, and space available for regeneration [9,16,33,34]. Taken together, these advantages contributed to the ideal condition for periodontal regeneration. Because of the above extraordinary advantages, extra additives may only express slim or even negligible effects. (3) Additional significant clinical benefits need longitudinal estimation after regenerative techniques are applied to the defects. The follow-up visit at 6 or 12 months might not be enough. (4) The actual regeneration impact of EMD and the application of guided tissue regeneration (GTR) were considerably variable [3,12,29,35–38]. It is difficult to obtain a predictable and reliable reconstruction of periodontal tissue by GTR, as there was a substantial variation in clinical response [3,39]. (5) MIS with recombinant human platelet-derived growth factor–BB gel alone might not provide an obviously additional benefit in the treatment of intra-bony defects without the concomitant use of osteoconductive biomaterials, such as β-TCP. (6) Hydroxyapatite (HA) biomaterial with GTR, potential obstruction of revascularization of the surgical site, could diminish the magnitude of SFA [16].

As far as we know, this is the first meta-analysis determining MIS plus regenerative biomaterials vs MIS alone in treating intra-bony defects. The included studies were all randomized, double-blinded, controlled clinical trials, so the results of this meta-analysis were trustworthy. Moreover, the heterogeneity of the included studies was low (I^2^ = 0). We described the low heterogeneity from the following two observations. All included studies underwent similar surgeries, and the surgeries had already produced optimal effects, so the extent of regeneration of intra-bony defects might be similar. The low heterogeneity might be attributed to merging of small sample sizes and the few available sample sizes. However, based on previous studies [15,40] and our clinical experience, we conducted a power and sample size calculation. Assuming an error of 5%, 80% power, expecting a SD of 1.5 mm and a difference of 1.8 mm in CAL change between the experiment and control groups, the results proved that a sample size of 12 patients per group would be needed. Hence, the sample size of each group in included studies was of significance.

Clearly, some limitations in our present study should be acknowledged. First, MIS, as a new technique in the periodontal field, lacks many primary studies. Moreover, we searched only literature published in English, which resulted in the limited sample sizes and the possibility that we missed some vital data that could have had a significant influence on the final results. In addition, reconstruction of the original PPD after periodontal surgery might be influenced by age, and patients with older age presented a lower increase in PPD [41]. Hence, a potential bias stemming from an uneven distribution of age for the subjects of the included studies should not be ignored. Finally, we did not perform a funnel plot because only four studies were included, so publication bias could not be estimated.

In view of the above results and limitations, we have some suggestions for further studies. It is important to conduct research to determine the inherent nature of the healing process of MIS. It is preferable to expand the sample size and perform comprehensive clinical trials for more reliable outcomes, if periodontal practitioners have performed analogous research, to confirm the results of our meta-analysis. Additionally, longer assessment durations should be performed to evaluate the effects of regenerative biomaterials. In addition, improved therapeutic outcomes and lower costs are common determinants that propel the acceptance of new technologies in clinical practice. Hence, the result of this meta-analysis reminds us that, from the economic and the beneficial points of view, costs and benefits should be taken into consideration. This consideration should be given to periodontal practitioners as well as patients when making decisions concerning a therapeutic approach to treat intra-bony defects.

In conclusion, the application of MIS provides prospective periodontal regeneration, and it poses several clinical advantages. This meta-analysis study indicated no additional benefits of MIS plus biomaterials in the treatment of intra-bony defects compared with MIS alone. Meanwhile, it presented evidence of a substantial clinical regeneration when MIS was applied in the treatment of intra-bony defects without regenerative biomaterials. It can be speculated that MIS will be popular in clinics and become a promising therapeutic modality to cure isolated or multiple periodontal intra-bony defects, replacing traditional surgical procedures in the future.

## Supporting Information

S1 PRISMA Checklist (DOCX)

S1 File Power and Sample size calculation. (DOCX)

S2 File A list of full-text excluded articles. (DOCX)
