# Supplementary material for: Minimally Invasive Surgery Combined with Regenerative Biomaterials in Treating Intra-Bony Defects: A Meta-Analysis
Source: PLoS One. 2016 Jan 19;11(1):e0147001. doi: 10.1371/journal.pone.0147001 (PMC4718618; doi:10.1371/journal.pone.0147001)
Supplement: S1 File — (DOCX) [file pone.0147001.s002.docx]

**Power and sample size calculation**

Because of the small sample size of each study we included, we conducted a sample size calculation using Power and Sample Size Calculations (PS, Version 3.0) software. We determined the standard deviation（SD）and tolerance error through extensive literatures searching and our clinical experiences. Besides, after calculation, we searched relevant literatures again to confirm the accuracy of our results.

Firstly, considering clinical attachment level (CAL) gain is the most pivotal outcome and it’s a gold standard to judge the success of periodontal treatment, so we conducted a sample size calculation regarding CAL gain.

The processes and results are as follows. Assuming an α error of 5%, 80% power, expecting a SD of 1.5mm and a difference of 1.8mm in clinical attachment level(CAL) change between the test and control groups, the result of sample size is 12 subjects per group.

According to our sample size calculation, we considered 12 subjects per group were needed. And then, we conducted the power analysis of each study we included to examine whether the number of patients per study is enough. The processes and results are as follows. Assuming an α error of 5%, an SD of 1.5mm, expecting a difference of 1.8mm in CAL change between the test and the control groups. Studies of Mishra and Trombelli respectively having a total sample size of 12 subjects per group were estimated to have a power of 80.2% to detect the difference between two groups. As for Ribeiro’s and Cortellini ＆Tonetti’s studies who respectively having a sample size of 15 subjects per group, were estimated to have 88.7% power to detect the difference between two groups.

Secondly, we searched relevant literatures concerning minimally invasive surgery treating intra-bony diseases again to affirm our power and sample size calculation is rational.

We list these relevant literatures as follows.

1.Howell TH, Fiorellini JP, Paquette DW, Offenbacher S, Giannobile WV, et al. (1997) A phase I/II clinical trial to evaluate a combination of recombinant human platelet-derived growth factor-BB and recombinant human insulin-like growth factor-I in patients with periodontal disease. J Periodontol 68: 1186-1193.

2. Cortellini P, Nieri M, Prato GP, Tonetti MS (2008) Single minimally invasive surgical technique with an enamel matrix derivative to treat multiple adjacent intra-bony defects: clinical outcomes and patient morbidity. J Clin Periodontol 35: 605-613.

3. Cortellini P, Tonetti MS (2007) Minimally invasive surgical technique and enamel matrix derivative in intra-bony defects. I: Clinical outcomes and morbidity. J Clin Periodontol 34: 1082-1088.

4. Cortellini P, Tonetti MS (2009) Improved wound stability with a modified minimally invasive surgical technique in the regenerative treatment of isolated interdental intrabony defects. J Clin Periodontol 36: 157-163.

5. Cortellini P, Tonetti MS (2011) Clinical and radiographic outcomes of the modified minimally invasive surgical technique with and without regenerative materials: a randomized-controlled trial in intra-bony defects. J Clin Periodontol 38: 365-373.

6. Harrel SK, Wilson TG, Jr., Nunn ME (2010) Prospective assessment of the use of enamel matrix derivative with minimally invasive surgery: 6-year results. J Periodontol 81: 435-441.

7. Mishra A, Avula H, Pathakota KR, Avula J (2013) Efficacy of modified minimally invasive surgical technique in the treatment of human intrabony defects with or without use of rhPDGF-BB gel: a randomized controlled trial. J Clin Periodontol 40: 172-179.

8. Ribeiro FV, Casarin RC, Junior FH, Sallum EA, Casati MZ (2011) The role of enamel matrix derivative protein in minimally invasive surgery in treating intrabony defects in single-rooted teeth: a randomized clinical trial. J Periodontol 82: 522-532.

9. Ribeiro FV, Casarin RC, Palma MA, Junior FH, Sallum EA, et al. (2011) Clinical and patient-centered outcomes after minimally invasive non-surgical or surgical approaches for the treatment of intrabony defects: a randomized clinical trial. J Periodontol 82: 1256-1266.

10. Ribeiro FV, Nociti Junior FH, Sallum EA, Sallum AW, Casati MZ (2010) Use of enamel matrix protein derivative with minimally invasive surgical approach in intra-bony periodontal defects: clinical and patient-centered outcomes. Braz Dent J 21: 60-67.

11. Trombelli L, Simonelli A, Pramstraller M, Wikesjo UM, Farina R (2010) Single flap approach with and without guided tissue regeneration and a hydroxyapatite biomaterial in the management of intraosseous periodontal defects. J Periodontol 81: 1256-1263.

12. Trombelli L, Simonelli A, Schincaglia GP, Cucchi A, Farina R (2012) Single-flap approach for surgical debridement of deep intraosseous defects: a randomized controlled trial. J Periodontol 83: 27-35.
