# Supplementary material for: Minimally Invasive Surgery Combined with Regenerative Biomaterials in Treating Intra-Bony Defects: A Meta-Analysis
Source: PLoS One. 2016 Jan 19;11(1):e0147001. doi: 10.1371/journal.pone.0147001 (PMC4718618; doi:10.1371/journal.pone.0147001)
Supplement: S2 File — (DOCX) [file pone.0147001.s003.docx]

**A list of full-text excluded articles with the reasons for exclusion**

Through primary database search and hand search, 464 articles were selected. Then, we deleted 298 articles because of duplication and some articles repeated twice or three times, so the sum of the duplicated articles was 166. Here we list excluded studies as follows [[1-166](#_ENREF_1)].

After excluding 298 duplicates, 154 articles removed by screening titles and abstracts [1-18, 23-47, 49-89,91-114,117,119-147,149-153,156-166].

Finally, 8 articles were excluded after full–test browsed [[19-21](#_ENREF_19),[48](#_ENREF_48),[116](#_ENREF_116),[118](#_ENREF_118),[148](#_ENREF_148),[155](#_ENREF_155)] and 4 articles according with the inclusion criteria were adopted[[22](#_ENREF_22),[90](#_ENREF_90),[115](#_ENREF_115),[154](#_ENREF_154)].

1. Arnott G, Merlen JF, Benoit M, Coget J, Fourlinnie JC, et al. (1972) [Gingiva in latent diabetes and subclinical diabetes (apropos of 4 personal cases)]. Lille Med 17: 1496-1505.

2. Avradopoulos V, Wilder RS, Chichester S, Offenbacher S (2004) Clinical and inflammatory evaluation of Perioscopy on patients with chronic periodontitis. J Dent Hyg 78: 30-38.

3. Banoczy J (2001) [Perspectives of dentistry in the 21st century]. Fogorv Sz 94: 3-7.

4. Baumann A, Ewers R (1999) [Minimally invasive sinus lift. Limits and possibilities in the atrophic maxilla]. Mund Kiefer Gesichtschir 3 Suppl 1: S70-73.

5. Becker W, Gabitov I, Stepanov M, Kois J, Smidt A, et al. (2010) Minimally invasive treatment for papillae deficiencies in the esthetic zone: a pilot study. Clin Implant Dent Relat Res 12: 1-8.

6. Becker W, Goldstein M, Becker BE, Sennerby L (2005) Minimally invasive flapless implant surgery: a prospective multicenter study. Clin Implant Dent Relat Res 7 Suppl 1: S21-27.

7. Becker W, Goldstein M, Becker BE, Sennerby L, Kois D, et al. (2009) Minimally invasive flapless implant placement: follow-up results from a multicenter study. J Periodontol 80: 347-352.

8. Beltran V, Fuentes R, Engelke W (2012) Endoscopic visualization of anatomic structures as a support tool in oral surgery and implantology. J Oral Maxillofac Surg 70: e1-6.

9. Bittencourt S, Del Peloso Ribeiro E, Sallum EA, Nociti FH, Jr., Casati MZ (2012) Surgical microscope may enhance root coverage with subepithelial connective tissue graft: a randomized-controlled clinical trial. J Periodontol 83: 721-730.

10. Bousdras V, Aghabeigi B, Hopper C, Sindet-Pedersen S (2006) Management of apical bone loss around a mandibular implant: a case report. Int J Oral Maxillofac Implants 21: 439-444.

11. Burkhardt R, Hurzeler MB (2000) Utilization of the surgical microscope for advanced plastic periodontal surgery. Pract Periodontics Aesthet Dent 12: 171-180; quiz 182.

12. Chaniotis A, Zervaki A (2013) Minimally invasive microsurgical management of the necrotic, immature apex tooth: case report and treatment recommendations. Quintessence Int 44: 429-436.

13. Chao JC (2012) A novel approach to root coverage: the pinhole surgical technique. Int J Periodontics Restorative Dent 32: 521-531.

14. Christensen GJ (2005) The advantages of minimally invasive dentistry. J Am Dent Assoc 136: 1563-1565.

15. Christensen GJ (2007) Current paradigm shifts in dentistry. Dent Today 26: 90, 92, 94 passim.

16. Cortellini P (2012) Minimally invasive surgical techniques in periodontal regeneration. J Evid Based Dent Pract 12: 89-100.

17. Cortellini P, Nieri M, Prato GP, Tonetti MS (2008) Single minimally invasive surgical technique with an enamel matrix derivative to treat multiple adjacent intra-bony defects: clinical outcomes and patient morbidity. J Clin Periodontol 35: 605-613.

18. Cortellini P, Pini-Prato G, Nieri M, Tonetti MS (2009) Minimally invasive surgical technique and enamel matrix derivative in intrabony defects: 2. Factors associated with healing outcomes. Int J Periodontics Restorative Dent 29: 257-265.

19. Cortellini P, Tonetti MS (2007) Minimally invasive surgical technique and enamel matrix derivative in intra-bony defects. I: Clinical outcomes and morbidity. J Clin Periodontol 34: 1082-1088.

20. Cortellini P, Tonetti MS (2007) A minimally invasive surgical technique with an enamel matrix derivative in the regenerative treatment of intra-bony defects: a novel approach to limit morbidity. J Clin Periodontol 34: 87-93.

21. Cortellini P, Tonetti MS (2009) Improved wound stability with a modified minimally invasive surgical technique in the regenerative treatment of isolated interdental intrabony defects. J Clin Periodontol 36: 157-163.

22. Cortellini P, Tonetti MS (2011) Clinical and radiographic outcomes of the modified minimally invasive surgical technique with and without regenerative materials: a randomized-controlled trial in intra-bony defects. J Clin Periodontol 38: 365-373.

23. Cosyn J, Cleymaet R, Hanselaer L, De Bruyn H (2012) Regenerative periodontal therapy of infrabony defects using minimally invasive surgery and a collagen-enriched bovine-derived xenograft: a 1-year prospective study on clinical and aesthetic outcome. J Clin Periodontol 39: 979-986.

24. Cosyn J, Eghbali A, Hanselaer L, De Rouck T, Wyn I, et al. (2013) Four modalities of single implant treatment in the anterior maxilla: a clinical, radiographic, and aesthetic evaluation. Clin Implant Dent Relat Res 15: 517-530.

25. Demir UL, Cetinkaya B, Karaca S, Sigirli D (2013) The impacts of adenotonsillar hypertrophy on periodontal health in children: a prospective controlled pilot study. Am J Otolaryngol 34: 501-504.

26. Dyer B, Sung EC (2012) Minimally Invasive Periodontal Treatment Using the Er,Cr: YSGG Laser. A 2-year Retrospective Preliminary Clinical Study. Open Dent J 6: 74-78.

27. Engelke W, Deckwer I (1997) Endoscopically controlled sinus floor augmentation. A preliminary report. Clin Oral Implants Res 8: 527-531.

28. Fermergard R, Astrand P (2008) Osteotome sinus floor elevation and simultaneous placement of implants--a 1-year retrospective study with Astra Tech implants. Clin Implant Dent Relat Res 10: 62-69.

29. Fermergard R, Astrand P (2012) Osteotome sinus floor elevation without bone grafts--a 3-year retrospective study with Astra Tech implants. Clin Implant Dent Relat Res 14: 198-205.

30. Filippi A, Meier ML, Lambrecht JT (2006) [Periradicular surgery with endoscopy--a clinical prospective study]. Schweiz Monatsschr Zahnmed 116: 12-17.

31. Franceschetti G, Farina R, Stacchi C, Di Lenarda R, Di Raimondo R, et al. (2014) Radiographic outcomes of transcrestal sinus floor elevation performed with a minimally invasive technique in smoker and non-smoker patients. Clin Oral Implants Res 25: 493-499.

32. Franceschetti G, Trombelli L, Minenna L, Farina R (2015) Learning Curve of a Minimally Invasive Technique for Transcrestal Sinus Floor Elevation: A Split-Group Analysis in a Prospective Case Series With Multiple Clinicians. Implant Dent.

33. Fu PY (2010) Piezoelectric-assisted osteotome-mediated sinus floor elevation: an innovative approach. Implant Dent 19: 299-306.

34. Gaitini L, Vaida S, Somri M, Croitoru M, Cherny N (1997) Tooth mobility--a risk management tool. Anaesthesia 52: 393-394.

35. Gaudio RM, Feltracco P, Barbieri S, Tiano L, Alberti M, et al. (2010) Traumatic dental injuries during anaesthesia: part I: clinical evaluation. Dent Traumatol 26: 459-465.

36. Gregg RH, 2nd, McCarthy D (2002) Laser periodontal therapy for bone regeneration. Dent Today 21: 54-59.

37. Grimm WD, Dannan A, Becher S, Gassmann G, Arnold W, et al. (2011) The ability of human periodontium-derived stem cells to regenerate periodontal tissues: a preliminary in vivo investigation. Int J Periodontics Restorative Dent 31: e94-e101.

38. Gupta SC, Jain S, Mehrotra R, Singh HP (2013) Central giant cell reparative granuloma of the ethmoids with bilateral proptosis and intracranial extension. Ear Nose Throat J 92: E6-9.

39. Hakim SG, Driemel O, Jacobsen HC, Hermes D, Sieg P (2006) Exposure of implants using a modified multiple-flap transposition vestibuloplasty. Br J Oral Maxillofac Surg 44: 507-510.

40. Han X, Liu H, Wang D, Su F, Zhang Y, et al. (2013) Alveolar bone regeneration around immediate implants using an injectable nHAC/CSH loaded with autogenic blood-acquired mesenchymal progenitor cells: an experimental study in the dog mandible. Clin Implant Dent Relat Res 15: 390-401.

41. Harnois P (2013) A new perspective on minimally invasive veneer techniques. Dent Today 32: 120, 124-125.

42. Harrel SK (1998) A minimally invasive surgical approach for periodontal bone grafting. Int J Periodontics Restorative Dent 18: 161-169.

43. Harrel SK (1999) A minimally invasive surgical approach for periodontal regeneration: surgical technique and observations. J Periodontol 70: 1547-1557.

44. Harrel SK, Abraham CM, Rivera-Hidalgo F, Shulman JD, Nunn ME (2014) Videoscope-assisted minimally invasive periodontal surgery (V-MIS). J Clin Periodontol 41: 900-907.

45. Harrel SK, Nunn ME, Belling CM (1999) Long-term results of a minimally invasive surgical approach for bone grafting. J Periodontol 70: 1558-1563.

46. Harrel SK, Rees TD (1995) Granulation tissue removal in routine and minimally invasive procedures. Compend Contin Educ Dent 16: 960, 962, 964 passim.

47. Harrel SK, Wilson TG, Nunn ME (2005) Prospective assessment of the use of enamel matrix proteins with minimally invasive surgery. J Periodontol 76: 380-384.

48. Harrel SK, Wilson TG, Jr., Nunn ME (2010) Prospective assessment of the use of enamel matrix derivative with minimally invasive surgery: 6-year results. J Periodontol 81: 435-441.

49. Harrel SK, Wilson TG, Jr., Rivera-Hidalgo F (2013) A videoscope for use in minimally invasive periodontal surgery. J Clin Periodontol 40: 868-874.

50. Harrel SK, Wright JM (2000) Treatment of periodontal destruction associated with a cemental tear using minimally invasive surgery. J Periodontol 71: 1761-1766.

51. Hauser F, Gaydarov N, Badoud I, Vazquez L, Bernard JP, et al. (2013) Clinical and histological evaluation of postextraction platelet-rich fibrin socket filling: a prospective randomized controlled study. Implant Dent 22: 295-303.

52. Hegde R, Sumanth S, Padhye A (2009) Microscope-enhanced periodontal therapy: a review and report of four cases. J Contemp Dent Pract 10: E088-096.

53. Held SA, Kao YH, Wells DW (1996) Endoscope--an endodontic application. J Endod 22: 327-329.

54. Hoskison E, Daniel M, Rowson JE, Jones NS (2012) Evidence of an increase in the incidence of odontogenic sinusitis over the last decade in the UK. J Laryngol Otol 126: 43-46.

55. Islam S, Loewenthal MR, Hoffman GR (2008) Use of peripherally inserted central catheters in the management of recalcitrant maxillofacial infection. J Oral Maxillofac Surg 66: 330-335.

56. Isler MS, Kolhatkar S, Bhola M (2008) Treatment of isolated recession defects using the lateral sliding flap: a case series. Pract Proced Aesthet Dent 20: 437-443; quiz 444, 432.

57. Iwai T, Tamai N, Matsui Y, Tohnai I (2012) Use of in-house, full-colour printed three-dimensional model for training in endoscopic periradicular surgery for molar radicular cyst. Br J Oral Maxillofac Surg 50: e41-42.

58. Jekl V, Hauptman K, Knotek Z (2008) Quantitative and qualitative assessments of intraoral lesions in 180 small herbivorous mammals. Vet Rec 162: 442-449.

59. Jeong SM, Choi BH, Li J, Xuan F (2008) Simultaneous flapless implant placement and peri-implant defect correction: an experimental pilot study in dogs. J Periodontol 79: 876-880.

60. Jofre J, Hamada T, Nishimura M, Klattenhoff C (2010) The effect of maximum bite force on marginal bone loss of mini-implants supporting a mandibular overdenture: a randomized controlled trial. Clin Oral Implants Res 21: 243-249.

61. Johansson CS, Ravald N, Pagonis C, Richter A (2014) Periodontitis in patients with coronary artery disease: an 8-year follow-up. J Periodontol 85: 417-425.

62. Julian J (2010) Extracting teeth with less trauma. Dent Today 29: 112-113.

63. Juodzbalys G, Bojarskas S, Kubilius R, Wang HL (2008) Using the support immersion endoscope for socket assessment. J Periodontol 79: 64-71.

64. Kaner D, Bernimoulin JP, Kleber BM, Friedmann A (2009) Minimally invasive flap surgery and enamel matrix derivative in the treatment of localized aggressive periodontitis: case report. Int J Periodontics Restorative Dent 29: 89-97.

65. Kawaguchi K, Amemiya T, Shimizu H, Hamada Y (2014) Image-guided robotic stereotactic radiotherapy for synchronous cancer of maxillary gingiva and lung. Int J Oral Maxillofac Surg 43: 692-695.

66. Kempe C, Dewes H, Gauer A, Stasche N (2001) [Peripheral giant cell reparative granuloma in the area of the nose floor. A review of current literature with a case report]. HNO 49: 1029-1033.

67. Kesler G (2004) Clinical applications of lasers during removable prosthetic reconstruction. Dent Clin North Am 48: 963-969, vii.

68. Kiyokawa K, Kiyokwa M, Sakaguchi S, Fukaya T, Rikimaru H (2009) Endoscopic maxillary sinus lift without vestibular mucosal incision or bone graft. J Craniofac Surg 20: 1462-1467.

69. Kiyokawa K, Rikimaru H, Kiyokawa M, Fukaya H, Sakaguchi S (2013) Treatment outcomes of implants performed after regenerative treatment of absorbed alveolar bone due to the severe periodontal disease and endoscopic surgery for maxillary sinus lift without bone grafts. J Craniofac Surg 24: 1599-1602.

70. Koutrach M, Nimmo A (2010) Preservation of existing soft-tissue contours in the transition from a tooth to an implant restoration in the esthetic zone using a flapless approach: a clinical report. J Prosthodont 19: 391-396.

71. Krekmanov L (2000) Placement of posterior mandibular and maxillary implants in patients with severe bone deficiency: a clinical report of procedure. Int J Oral Maxillofac Implants 15: 722-730.

72. Kurien T, Deo V, Bhati A (2010) The pouch and tunnel technique for the management of adjacent gingival recession defects: surgical correction and one-year follow-up. J Contemp Dent Pract 11: 041-048.

73. Kwan JY (2005) Enhanced periodontal debridement with the use of micro ultrasonic, periodontal endoscopy. J Calif Dent Assoc 33: 241-248.

74. Kwan JY (2006) The extreme cleaning makeover in dentistry. Interview by Lynne H Slim. Dent Today 25: 66, 68, 70.

75. Kwon DH, Bennett W, Herberg S, Bastone P, Pippig S, et al. (2010) Evaluation of an injectable rhGDF-5/PLGA construct for minimally invasive periodontal regenerative procedures: a histological study in the dog. J Clin Periodontol 37: 390-397.

76. Lamster IB, Smith QT, Celenti RS, Singer RE, Grbic JT (1994) Development of a risk profile for periodontal disease: microbial and host response factors. J Periodontol 65: 511-520.

77. Landsberg CJ (2008) Implementing socket seal surgery as a socket preservation technique for pontic site development: surgical steps revisited--a report of two cases. J Periodontol 79: 945-954.

78. Lazarovici TS, Yahalom R, Taicher S, Elad S, Hardan I, et al. (2009) Bisphosphonate-related osteonecrosis of the jaws: a single-center study of 101 patients. J Oral Maxillofac Surg 67: 850-855.

79. Lee CT, Hamalian T, Schulze-Spate U (2015) Minimally invasive treatment of soft tissue deficiency around an implant-supported restoration in the esthetic zone: modified VISTA technique case report. J Oral Implantol 41: 71-76.

80. Lee DH, Choi BH, Jeong SM, Xuan F, Kim HR (2011) Effects of flapless implant surgery on soft tissue profiles: a prospective clinical study. Clin Implant Dent Relat Res 13: 324-329.

81. Lee JY, Byun JY (2010) Huge radicular cyst. Otolaryngol Head Neck Surg 143: 704-705.

82. Liu F, Steinkeler A (2013) Epidemiology, diagnosis, and treatment of temporomandibular disorders. Dent Clin North Am 57: 465-479.

83. Longhini AB, Branstetter BF, Ferguson BJ (2010) Unrecognized odontogenic maxillary sinusitis: a cause of endoscopic sinus surgery failure. Am J Rhinol Allergy 24: 296-300.

84. Lopes N, Oliveira DM, Vajgel A, Pita I, Bezerra T, et al. (2009) A new approach for reconstruction of a severely atrophic mandible. J Oral Maxillofac Surg 67: 2455-2459.

85. Malterud MI (2013) Continuing education holds the key to minimally invasive biomimetic dental successes. Gen Dent 61: 8-11.

86. Mancini G, Buonaccorsi S, Reale G, Tedaldi M (2012) Application of piezoelectric device in endoscopic sinus surgery. J Craniofac Surg 23: 1736-1740.

87. Mandinic Z, Vulicevic ZR, Beloica M, Radovic I, Mandic J, et al. (2014) [The application of air abrasion in dentistry]. Srp Arh Celok Lek 142: 99-105.

88. McGuire MK, Scheyer ET (2007) A randomized, double-blind, placebo-controlled study to determine the safety and efficacy of cultured and expanded autologous fibroblast injections for the treatment of interdental papillary insufficiency associated with the papilla priming procedure. J Periodontol 78: 4-17.

89. Metzger Z, Huber R, Slavescu D, Dragomirescu D, Tobis I, et al. (2009) Healing kinetics of periapical lesions enhanced by the apexum procedure: a clinical trial. J Endod 35: 153-159.

90. Mishra A, Avula H, Pathakota KR, Avula J (2013) Efficacy of modified minimally invasive surgical technique in the treatment of human intrabony defects with or without use of rhPDGF-BB gel: a randomized controlled trial. J Clin Periodontol 40: 172-179.

91. Mitchell DM, Mitchell DN, Collins JV, Emerson CJ (1980) Transbronchial lung biopsy through fibreoptic bronchoscope in diagnosis of sarcoidosis. Br Med J 280: 679-681.

92. Moergel M, Walter C, Coerdt W, Reichert TE, Kunkel M (2004) [Chronic cutaneous infiltration with abscess and fistula formation. A type of clinical course in atypical mycobacteriosis]. Mund Kiefer Gesichtschir 8: 311-315.

93. Mombelli A, Decaillet F, Almaghlouth A, Wick P, Cionca N (2011) [Efficient, minimally invasive periodontal therapy. An evidence based treatment concept]. Schweiz Monatsschr Zahnmed 121: 145-157.

94. Montevecchi M, Checchi V, Bonetti GA (2012) Management of a deeply impacted mandibular third molar and associated large dentigerous cyst to avoid nerve injury and improve periodontal healing: case report. J Can Dent Assoc 78: c59.

95. Moshonov J, Michaeli E, Nahlieli O (2009) Endoscopic root canal treatment. Quintessence Int 40: 739-744.

96. Mourao J, Neto J, Luis C, Moreno C, Barbosa J, et al. (2013) Dental injury after conventional direct laryngoscopy: a prospective observational study. Anaesthesia 68: 1059-1065.

97. Nadjem H, Pollak S, Windisch W, Perdekamp MG, Thierauf A (2010) Tooth aspiration: its relevance in medicolegal autopsies. Forensic Sci Int 200: e25-29.

98. Nasr HF, de Nasr AM (1999) The semilunar flap technique for root coverage. Atlas Oral Maxillofac Surg Clin North Am 7: 29-37.

99. Nathwani NS, Kelleher M (2010) Minimally destructive management of amelogenesis imperfecta and hypodontia with bleaching and bonding. Dent Update 37: 170-172, 175-176, 179.

100. Nestal Zibo H, Miller E (2011) Endoscopically assisted enucleation of a large mandibular periapical cyst. Stomatologija 13: 128-131.

101. Nibali L (2014) Intrabony defects and non-surgical treatment. Prim Dent J 3: 48-50.

102. Ohba S, Yamashita H, Takashi I, Asahina I (2013) Marginal mandibulectomy for lower gingival carcinoma with a cheek-splitting transbuccal approach and reconstruction by buccal fat pad flap: a case report. J Oral Maxillofac Surg 71: e143-146.

103. Orekhova L, Neizberg DM, Stiuf I (2006) [Clinical-immunological and microbiological parallels in chronic generalized parodontitis and peptic ulcer of the stomach]. Stomatologiia (Mosk) 85: 22-26.

104. Ostertag P, Leunig A, Grevers G (1996) [Central (reparative) giant cell granuloma of the paranasal sinus]. Laryngorhinootologie 75: 619-622.

105. Ower P (2013) Minimally-invasive non-surgical periodontal therapy. Dent Update 40: 289-290, 293-285.

106. Ozawa T, Tsuchida M, Yamazaki Y, Arai T (2003) Minimally invasive periapical curettage of foreign materials in periapical lesions using a fiberscope. Int Dent J 53: 314-322.

107. Papaspyridakos P, White GS, Lal K (2012) Flapless CAD/CAM-guided surgery for staged transition from failing dentition to complete arch implant rehabilitation: a 3-year clinical report. J Prosthet Dent 107: 143-150.

108. Perkins JD, Windley Z, Dixon PM, Smith M, Barakzai SZ (2009) Sinoscopic treatment of rostral maxillary and ventral conchal sinusitis in 60 horses. Vet Surg 38: 613-619.

109. Pfohler C, Korner R, Vogt T, Muller CS (2012) Contact allergic gastritis: an underdiagnosed entity? BMJ Case Rep 2012.

110. Pinto RC, Chambrone L, Colombini BL, Ishikiriama SK, Britto IM, et al. (2013) Minimally invasive esthetic therapy: a case report describing the advantages of a multidisciplinary approach. Quintessence Int 44: 385-391.

111. Pozzi A, Sannino G, Barlattani A (2012) Minimally invasive treatment of the atrophic posterior maxilla: a proof-of-concept prospective study with a follow-up of between 36 and 54 months. J Prosthet Dent 108: 286-297.

112. Ramzan PH (2009) Oral endoscopy as an aid to diagnosis of equine cheek tooth infections in the absence of gross oral pathological changes: 17 cases. Equine Vet J 41: 101-106.

113. Re M, Zizzi A, Aspriello SD, Stramazzotti D, Rubini C, et al. (2011) Videoendoscopic assisted curettage of central giant cell granuloma of the maxilla in pediatric age. Minerva Stomatol 60: 321-325.

114. Rethman MP, Harrel SK (2010) Minimally invasive periodontal therapy: will periodontal therapy remain a technologic laggard? J Periodontol 81: 1390-1395.

115. Ribeiro FV, Casarin RC, Junior FH, Sallum EA, Casati MZ (2011) The role of enamel matrix derivative protein in minimally invasive surgery in treating intrabony defects in single-rooted teeth: a randomized clinical trial. J Periodontol 82: 522-532.

116. Ribeiro FV, Casarin RC, Palma MA, Junior FH, Sallum EA, et al. (2011) Clinical and patient-centered outcomes after minimally invasive non-surgical or surgical approaches for the treatment of intrabony defects: a randomized clinical trial. J Periodontol 82: 1256-1266.

117. Ribeiro FV, Hirata DY, Reis AF, Santos VR, Miranda TS, et al. (2014) Open-flap versus flapless esthetic crown lengthening: 12-month clinical outcomes of a randomized controlled clinical trial. J Periodontol 85: 536-544.

118. Ribeiro FV, Nociti Junior FH, Sallum EA, Sallum AW, Casati MZ (2010) Use of enamel matrix protein derivative with minimally invasive surgical approach in intra-bony periodontal defects: clinical and patient-centered outcomes. Braz Dent J 21: 60-67.

119. Sagit M, Guler S, Tasdemir A, Akf Somdas M (2011) Large radicular cyst in the maxillary sinus. J Craniofac Surg 22: e64-65.

120. Salazar CR, Francois F, Li Y, Corby P, Hays R, et al. (2012) Association between oral health and gastric precancerous lesions. Carcinogenesis 33: 399-403.

121. Scheyer ET (2003) Periodontal endoscopy in clinical practice. Pract Proced Aesthet Dent 15: 36-39.

122. Schulz M, Bosshardt D, von Arx T (2009) [Periapical surgery with histologic examination of the periapical lesion. A case report]. Schweiz Monatsschr Zahnmed 119: 991-1005.

123. Seefelder C, Ko JH, Padwa BL (2000) Fibreoptic intubation for massive gingival hyperplasia in juvenile hyaline fibromatosis. Paediatr Anaesth 10: 682-684.

124. Selden HS (1999) A conservative biopsy technique for periapical lesions. J Endod 25: 769-770.

125. Seno S, Ogawal T, Shibayama M, Ogawa F, Fukui J, et al. (2009) Endoscopic sinus surgery for the odontogenic maxillary cysts. Rhinology 47: 305-309.

126. Seow WK, Perham S, Young WG, Daley T (1990) Dilaceration of a primary maxillary incisor associated with neonatal laryngoscopy. Pediatr Dent 12: 321-324.

127. Seydel HG, Scholl H (1973) Permanent implants in the management of head and neck cancer by radiotherapy. Am J Roentgenol Radium Ther Nucl Med 117: 565-574.

128. Shahbazian M, Jacobs R, Wyatt J, Denys D, Lambrichts I, et al. (2013) Validation of the cone beam computed tomography-based stereolithographic surgical guide aiding autotransplantation of teeth: clinical case-control study. Oral Surg Oral Med Oral Pathol Oral Radiol 115: 667-675.

129. Sharma R, Hegde V, Siddharth M, Hegde R, Manchanda G, et al. (2014) Endodontic-periodontal microsurgery for combined endodontic-periodontal lesions: An overview. J Conserv Dent 17: 510-516.

130. Shumrick DA, Quenelle DJ (1979) Malignant disease of the tonsillar region, retromolar trigone, and buccal mucosa. Otolaryngol Clin North Am 12: 115-124.

131. Silva Rossi-Aguiar VP, Navarro-Rodriguez T, Mattar R, Siqueira de Melo Peres MP, Correa Barbuti R, et al. (2009) Oral cavity is not a reservoir for Helicobacter pylori in infected patients with functional dyspepsia. Oral Microbiol Immunol 24: 255-259.

132. Singhal SK, Chhabra B (1996) Loose tooth: a problem. Anesth Analg 83: 1352.

133. Sipes JN, Thompson RL, Hook EW (1977) Prophylaxis of infective endocarditis: a reevaluation. Annu Rev Med 28: 371-391.

134. Sipkin AM, Nikitin AA, Kekukh EO (2011) [The treatment and rehabilitation of patients with secondary adentia and atrophy of alveolar process of maxilla]. Khirurgiia (Mosk): 54-57.

135. Smith LP, Rose T (2010) Laser explantation of a failing endosseous dental implant. Aust Dent J 55: 219-222.

136. Soardi E, Cosci F, Checchi V, Pellegrino G, Bozzoli P, et al. (2013) Radiographic analysis of a transalveolar sinus-lift technique: a multipractice retrospective study with a mean follow-up of 5 years. J Periodontol 84: 1039-1047.

137. Sperandio FF, Carli ML, Guimaraes EP, Pereira AA, Hanemann JA (2014) Noninvasive treatment choice for an aged down syndrome patient presenting a residual periapical cyst. J Contemp Dent Pract 15: 254-257.

138. Stambaugh RV (2002) A clinician's 3-year experience with perioscopy. Compend Contin Educ Dent 23: 1061-1070.

139. Stambaugh RV, Myers G, Ebling W, Beckman B, Stambaugh K (2002) Endoscopic visualization of the submarginal gingiva dental sulcus and tooth root surfaces. J Periodontol 73: 374-382.

140. Struch F, Schwahn C, Wallaschofski H, Grabe HJ, Volzke H, et al. (2008) Self-reported halitosis and gastro-esophageal reflux disease in the general population. J Gen Intern Med 23: 260-266.

141. Szabo G, Miko I, Peto K, Brath E, Nagy P, et al. (2005) [Laparoscopic versus open cholecystectomy: reaction in the liver bed]. Magy Seb 58: 106-110.

142. Takano JH, Yakushiji T, Kamiyama I, Nomura T, Katakura A, et al. (2010) Detecting early oral cancer: narrowband imaging system observation of the oral mucosa microvasculature. Int J Oral Maxillofac Surg 39: 208-213.

143. Taschieri S, Del Fabbro M, Testori T, Francetti L, Weinstein R (2006) Endodontic surgery using 2 different magnification devices: preliminary results of a randomized controlled study. J Oral Maxillofac Surg 64: 235-242.

144. Taschieri S, Del Fabbro M, Testori T, Weinstein R (2007) Endoscopic periradicular surgery: a prospective clinical study. Br J Oral Maxillofac Surg 45: 242-244.

145. Taschieri S, Fabbro MD, Corbella S, Weinstein T, Rosano G, et al. (2011) Endoscopic minimally invasive management of a periradicular lesion invading the maxillary sinus. J Oral Sci 53: 533-538.

146. Taschieri S, Rosano G, Weinstein T, Del Fabbro M (2008) Endoscopic management of a lateral root lesion. A case report. Minerva Stomatol 57: 587-595.

147. Testori T, Mandelli F, Mantovani M, Taschieri S, Weinstein RL, et al. (2013) Tilted trans-sinus implants for the treatment of maxillary atrophy: case series of 35 consecutive patients. J Oral Maxillofac Surg 71: 1187-1194.

148. Trombelli L, Farina R, Franceschetti G, Calura G (2009) Single-flap approach with buccal access in periodontal reconstructive procedures. J Periodontol 80: 353-360.

149. Trombelli L, Franceschetti G, Rizzi A, Minenna P, Minenna L, et al. (2012) Minimally invasive transcrestal sinus floor elevation with graft biomaterials. A randomized clinical trial. Clin Oral Implants Res 23: 424-432.

150. Trombelli L, Franceschetti G, Stacchi C, Minenna L, Riccardi O, et al. (2014) Minimally invasive transcrestal sinus floor elevation with deproteinized bovine bone or beta-tricalcium phosphate: a multicenter, double-blind, randomized, controlled clinical trial. J Clin Periodontol 41: 311-319.

151. Trombelli L, Franceschetti G, Trisi P, Farina R (2015) Incremental, transcrestal sinus floor elevation with a minimally invasive technique in the rehabilitation of severe maxillary atrophy. Clinical and histological findings from a proof-of-concept case series. J Oral Maxillofac Surg 73: 861-888.

152. Trombelli L, Minenna P, Franceschetti G, Minenna L, Farina R (2010) Transcrestal sinus floor elevation with a minimally invasive technique. J Periodontol 81: 158-166.

153. Trombelli L, Minenna P, Franceschetti G, Minenna L, Itro A, et al. (2010) Minimally invasive technique for transcrestal sinus floor elevation: a case report. Quintessence Int 41: 363-369.

154. Trombelli L, Simonelli A, Pramstraller M, Wikesjo UM, Farina R (2010) Single flap approach with and without guided tissue regeneration and a hydroxyapatite biomaterial in the management of intraosseous periodontal defects. J Periodontol 81: 1256-1263.

155. Trombelli L, Simonelli A, Schincaglia GP, Cucchi A, Farina R (2012) Single-flap approach for surgical debridement of deep intraosseous defects: a randomized controlled trial. J Periodontol 83: 27-35.

156. Vaderhobli RM, White JM, Le C, Ho S, Jordan R (2010) In vitro study of the soft tissue effects of microsecond-pulsed CO(2) laser parameters during soft tissue incision and sulcular debridement. Lasers Surg Med 42: 257-263.

157. von Arx T, Montagne D, Zwinggi C, Lussi A (2003) Diagnostic accuracy of endoscopy in periradicular surgery - a comparison with scanning electron microscopy. Int Endod J 36: 691-699.

158. Watanabe T, Marchack BW, Takei HH (2013) Creating labial bone for immediate implant placement: a minimally invasive approach by using orthodontic therapy in the esthetic zone. J Prosthet Dent 110: 435-441.

159. Widera D, Grimm WD, Moebius JM, Mikenberg I, Piechaczek C, et al. (2007) Highly efficient neural differentiation of human somatic stem cells, isolated by minimally invasive periodontal surgery. Stem Cells Dev 16: 447-460.

160. Wilson TG, Jr., Carnio J, Schenk R, Myers G (2008) Absence of histologic signs of chronic inflammation following closed subgingival scaling and root planing using the dental endoscope: human biopsies - a pilot study. J Periodontol 79: 2036-2041.

161. Wilson TG, Harrel SK, Nunn ME, Francis B, Webb K (2008) The relationship between the presence of tooth-borne subgingival deposits and inflammation found with a dental endoscope. J Periodontol 79: 2029-2035.

162. Yamada Y, Hara K, Nakamura S, Ueda M, Ito K, et al. (2013) Minimally invasive approach with tissue engineering for severe alveolar bone atrophy case. Int J Oral Maxillofac Surg 42: 260-263.

163. Young GR (2013) Endoscopic periodontal debridement. Dent Today 32: 122-125.

164. Zadeh HH (2011) Minimally invasive treatment of maxillary anterior gingival recession defects by vestibular incision subperiosteal tunnel access and platelet-derived growth factor BB. Int J Periodontics Restorative Dent 31: 653-660.

165. Zadeh HH, Daftary F (2004) Minimally invasive surgery: an alternative approach for periodontal and implant reconstruction. J Calif Dent Assoc 32: 1022-1030.

166. Zingale J, Harpenau L, Chambers D, Lundergan W (2012) Effectiveness of root planing with diode laser curettage for the treatment of periodontitis. J Calif Dent Assoc 40: 786-793.
